# Progress towards the implementation of control programmes for strongyloidiasis in endemic areas: estimation of number of adults in need of ivermectin for strongyloidiasis

**DOI:** 10.1098/rstb.2022.0433

**Published:** 2024-01-15

**Authors:** Dora Buonfrate, Antonio Montresor, Zeno Bisoffi, Francesca Tamarozzi, Donal Bisanzio

**Affiliations:** ^1^ Department of Infectious Tropical Diseases and Microbiology, IRCCS Sacro Cuore don Calabria Hospital, 370242, Negrar, Verona, Italy; ^2^ Department of Control of Neglected Tropical Diseases, World Health Organization, 12024, Geneva, Switzerland; ^3^ Research Triangle Institute International, Washington, DC 20005-3967, USA; ^4^ Division of Epidemiology and Public Health, School of Medicine, University of Nottingham, Nottingham, NG5 1PB, UK

**Keywords:** *Strongyloides*, World Health Organization, preventive chemotherapy, mass drug administration, ivermectin

## Abstract

The World Health Organization has started a process to issue guidelines for the control of strongyloidiasis. The guidelines might recommend to implement preventive chemotherapy (PC) at community level (i.e. to all individuals above 5 years of age), over a defined prevalence threshold. We previously estimated the number of school-age children (SAC) who would need PC. Here we estimate the number of people above 15 years of age who might be included in PC for strongyloidiasis. Based on previous *Strongyloides* prevalence estimates and on countries' age distribution, we retrieved the number of adults in need of PC. We then subtracted the number of people already involved in ivermectin mass distribution for the elimination of onchocerciasis and lymphatic filariasis and people living in countries where *Loa loa* is endemic. The number of adults to be involved in PC was estimated at 905.4 (95% confidence interval (CI): 520.6–1177.2), 660.2 (95% CI: 512.7-1214.9), and 512.1 (95% CI: 276–719.4) million people, when the strongyloidiasis prevalence threshold for implementing PC was set to 10%, 15% and 20%, respectively. Estimates at country level are also provided.These estimates might help endemic countries wishing to implement PC for strongyloidiasis to allocate resources to include adults in addition to SAC in control programmes.

This article is part of the Theo Murphy meeting issue ‘*Strongyloides*: omics to worm-free populations’.

## 1. Introduction

The World Health Organization (WHO) 2030 targets for the control of soil-transmitted helminths (STH) in endemic areas included, for the first time, the recommendation for implementation of control activities for *Strongyloides stercoralis* infection in school-age children (SAC) [1]. The integration of ongoing programmes for the other STH with specific activities targeting strongyloidiasis was envisaged in that document, as common infrastructures and staff would permit to reduce costs and ease the implementation, despite the need for a different diagnostic approach (Kato-Katz, which is used for prevalence assessment of the other STH, has an extremely low sensitivity for the detection of *Strongyloides* larvae) and medication (ivermectin (IVM) instead of benzimidazoles) [2,3]. Overall, there is need for guidance from the WHO in order to harmonize and set up a proper strategy. The process towards the issuing of WHO guidelines has started recently, with the formation of a Guidelines Developing Group (GDG), a group of experts responsible for evaluating the existing evidence and, if warranted, to recommend control interventions. The first task of the GDG was to collect evidence about key points that constitute the basis of the guidelines. These key points include: (i) global prevalence of strongyloidiasis; (ii) morbidity and mortality caused by the infection; (iii) accuracy of diagnostic tests for diagnosis at population level; (iv) efficacy and safety of IVM; (v) impact of large-scale distribution of IVM; (vi) risks for non -infected to be treated; (vii) cost of IVM distribution versus cost of disease; and (viii) benefits for other parasitoses.

While the document reporting the 2030 targets promoted the implementation of control activities for strongyloidiasis in SAC, the evidence collected by the GDG, supported by a modelling study, seems to shift the focus of control strategies at the whole-age community level. One main reason for this is the higher prevalence of infection in adults than children, owing to chronic infection, persisting indefinitely if not treated, as the consequence of the peculiar auto-infective cycle of the parasite. Although studies reporting data on age distribution of strongyloidiasis are scant [4–8], the higher prevalence in adults is biologically plausible, since in older ages we have a cumulative rate of strongyloidiasis owing to chronic and new infections that occur over time.

Preventive chemotherapy (PC) is the main pillar of the WHO control strategies for STH [2], therefore the evidence retrieved by the GDG will be used by modellers to identify prevalence thresholds above which PC is recommended, and the frequency (annual/biannual) of PC rounds. If a community-level approach will be confirmed in the WHO guidelines, the PC strategy recommended would be mass drug administration (MDA) to people of all age groups (excluding children under 5 years of age, for whom IVM is not approved [9]).

MDA at community level, as implemented for example for the control of filarial infections [2,10,11], would have higher costs, because of the need to establish a distribution infrastructure (that in the case of children and STH is already present, actualized in the school-based distribution strategy), but would presumably impact the outcome of the intervention, with a sharper decrease of the prevalence of *S. stercoralis* infection in the target community [12].

To plan such community-wide MDA, it is fundamental to provide estimates of the magnitude of the population that would need to be covered by a possible PC intervention. This would help the countries that wish to implement PC for strongyloidiasis to allocate resources. In a previous work, we estimated the number of SAC that would need PC for strongyloidiasis, in line with the previous envisaged target [13].

Here, we report the estimates of the number of people over 15 years of age who would be eligible for PC for strongyloidiasis, at global and country level.

## 2. Methods

The study was based on national-level prevalence of strongyloidiasis estimated previously for the year 2017 [8]. Given that endemic countries have not deployed large-scale programmes targeting strongyloidiasis, we assume that the 2017 estimated prevalence should not have changed significantly during the last five years. Demographic data of included countries were retrieved from the World Bank Open Data website [14]. The gathered data were the adult population (people older than 15 years of age) proportions living in rural areas for the year 2022. MDA with IVM is provided in campaigns targeting lymphatic filariasis (LF) or onchocerciasis (OV); though the dosage in these programmes may vary from 150 to 400 µg kg^−1^ (for strongyloidiasis being 200 µg kg^−1^) [9–11], we believe that these areas would not deserve additional rounds of IVM for strongyloidiasis. Hence, we retrieved from the WHO the number of people who received IVM in elimination programmes for these filarial infections between 2000 and 2018 [15], to exclude them from the number of people who should be included in mass treatment for strongyloidiasis. We arbitrarily chose three different strongyloidiasis prevalence thresholds as possible PC-trigging thresholds (10%, 15% and 20%) that might be recommended to start implementing PC. Based on each threshold, we calculated the number of people to be covered by national MDA campaign.

As performed previously, we estimated the prevalence of strongyloidiasis at subnational level using the normal approximation of the binomial distribution [13]. Using countries’ age distribution obtained from the World Bank, we estimated the number of adults to be covered by PC. We also assumed that if the prevalence was zero, nobody would meet the threshold to trigger PC. Thus, we created estimates of the population needing to be covered by PC for the three intervention thresholds. Using a linear model, we built a polynomial function to estimate the adult population living in areas with prevalence above the PC thresholds.

The estimate of adults to be treated with PC was then adjusted by taking into account the fraction of people who may already have been treated through previous elimination programmes for LF and OV (data retrieved through the WHO Global Health Observatory and PCT Databank [15,16]) and those who live in areas with *Loa loa* circulation (who should not receive IVM, based on the risk of developing severe encephalopathy if harbouring high microfilarial load) [17]. While the data on OV MDA provide the number of adults treated, the LF PC data do not provide the number of treatments broken down by age group [15]. Therefore, to estimate the number of adults already covered by LF MDA, we divided the number of treated people using the countries' age-group distribution; we assume that LF MDA equally covered all age groups in any given country. The estimates of adults requiring treatment with IVM taking in account *L. loa* presence was performed by using the estimates adjusted by LF/OV MDA and removing all countries in which *L. loa* is endemic. The list of *L. loa* endemic countries was based on the map published by Zoure *et al*. [18]. As we were not able to extrapolate the map's high-resolution data, we decided to remove from our estimates all adults living in *L. loa* endemic countries.

## 3. Results

We first estimated the number of adults to be covered by PC for strongyloidiasis at country level, not considering those already included in IVM MDA for filarial infections. Estimates are reported in the electronic supplementary material, tables S1-S3. The results of our analyses showed that the number of adults that should be covered with PC campaigns (using 2022 population data) is 994.2 million (95% confidence interval (CI): 571.6–1292.6), 725 million (95% CI: 563.1–1333.9), and 562.3 million (95% CI: 303.1–790.1) when the strongyloidiasis prevalence threshold to trigger PC (PTPC) was set to 10%, 15% and 20%, respectively (table 1). The adult population to be covered with PC in the WHO southeast Asia region (SEAR) accounted for approximately 50% (PTPC 10%: 499.5 million (95% CI: 291.9–635.6); PTPC 15%: 367.6 million (95% CI: 288.1–662.9); PTPC 20%: 287.2 million (95% CI: 155.9–400.4)) of all adults that should be targeted by PC for the three intervention thresholds (table 1). The Africa region (AFR) and western Pacific region (WPR) followed in terms of estimated number of adults needing to be treated with PC, with figures about one third of the SEAR's estimates (table 1).

**Table 1.** Estimates of the number of adults that would be included in PC, at different thresholds of prevalence of strongyloidiasis. (The estimates accounted for IVM MDA for LF/OV and for *Loa loa* endemicity. STG, *Strongyloides*; PC, preventive chemotherapy; MDA, mass drug administration; LF, lymphatic filariasis; OV, *Onchocerca volvulus*; 95% CI, 95% confidence interval; AFR, African region; AMR, American region; EMR, eastern Mediterranean region; SEAR, southeast Asian region; WPR, western Pacific region; EUR, European region.)

WHO regions	STG-PC adult population (million (95% CI))	STG-PC adult population (million (95% CI)) not covered by LF/OV-PC	STG-PC adult population (million (95% CI)) not covered by LF/OV-PC and not living in *Loa loa* endemic countries
threshold: 10% prevalence			
AFR	193.4 (110.6–253.1)	141.8 (80.9–186.2)	104.9 (59.7–138.1)
AMR	43 (25–55.1)	43 (25–55.1)	43 (25–55.1)
EMR	55.6 (30.4–76.8)	55.3 (30.3–76.4)	55.3 (30.3–76.4)
EUR	6.4 (3.5–8.9)	6.4 (3.5–8.9)	6.4 (3.5–8.9)
SEAR	499.5 (291.9–635.6)	499.5 (291.9–635.6)	499.5 (291.9–635.6)
WPR	196.3 (110.2–263.1)	196.3 (110.2–263.1)	196.3 (110.2–263.1)
World	994.2 (571.6–1292.6)	942.3 (541.8–1225.3)	905.4 (520.6 1177.2)
threshold: 15% prevalence			
AFR	140.6 (108.9–260.3)	102.9 (79.6–191.2)	76.1 (58.7–141.7)
AMR	31.6 (24.7–57.3)	31.6 (24.7–57.3)	31.6 (24.7–57.3)
EMR	39.5 (29.8–77)	39.2 (29.6–76.6)	39.2 (29.6–76.6)
EUR	4.5 (3.4–8.9)	4.5 (3.4–8.9)	4.5 (3.4–8.9)
SEAR	367.6 (288.1–662.9)	367.6 (288.1–662.9)	367.6 (288.1–662.9)
WPR	141.2 (108.2–267.5)	141.2 (108.2–267.5)	141.2 (108.2–267.5)
World	725 (563.1–1333.9)	687 (533.6–1264.4)	660.2 (512.7–1214.9)
threshold: 20% prevalence			
AFR	108.8 (58.5–153.3)	79.6 (42.7–112.2)	58.7 (31.5–82.9)
AMR	24.6 (13.3–34.4)	24.6 (13.3–34.4)	24.6 (13.3–34.4)
EMR	29.9 (15.8–43.1)	29.8 (15.7–42.8)	29.8 (15.7–42.8)
EUR	3.4 (1.8–4.9)	3.4 (1.8–4.9)	3.4 (1.8–4.9)
SEAR	287.2 (155.9–400.4)	287.2 (155.9–400.4)	287.2 (155.9–400.4)
WPR	108.4 (57.8–154)	108.4 (57.8–154)	108.4 (57.8–154)
World	562.3 (303.1–790.1)	533 (287.2–748.7)	512.1 (276–719.4)

**Figure 1.**
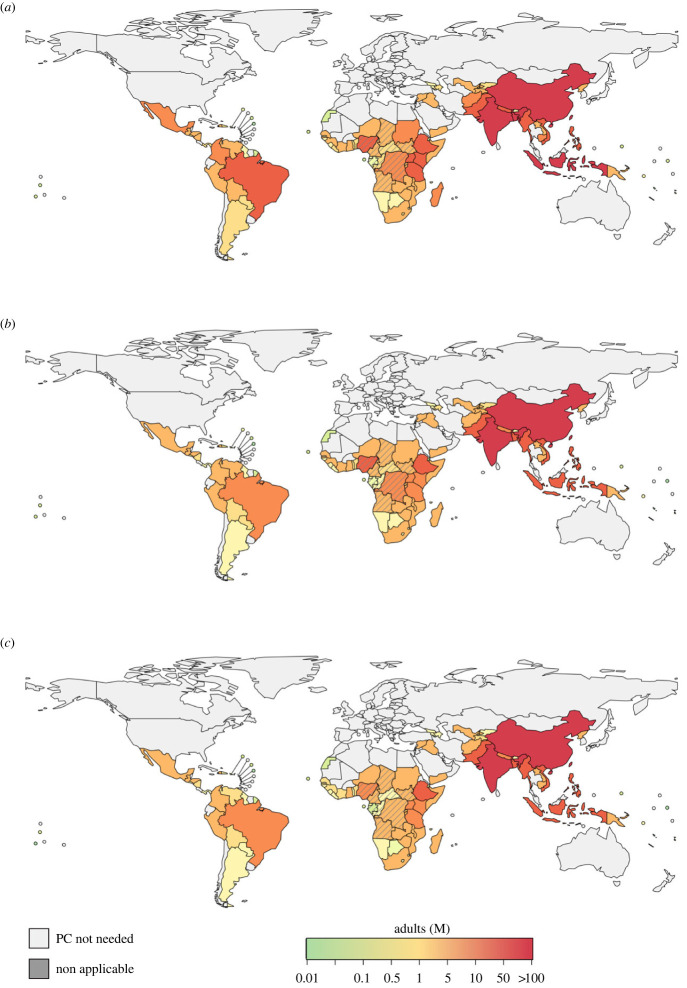
Estimated number of adults (older than 15 years of age) to be enrolled in PC at country level, when the strongyloidiasis prevalence threshold to start the intervention was set to (*a*) 10%, (*b*) 15% and (*c*) 20%. The map only shows estimates for countries for which WHO has recommended PC for other STHs. The estimates take into account IVM MDA from LF and OV. Barred countries are *Loa loa* endemic.

Among all selected countries, India, China, Indonesia, Bangladesh, Nigeria, and Pakistan accounted for more than 50% of global adult population that should be targeted by PC at the three selected intervention thresholds (figure 1; electronic supplementary material, tables S1–S3).

We then estimated the population to be covered with PC accounting for the proportion of the population already included in previous MDA campaigns targeting LF and OV between 2013 and 2018. This led to a reduced number of people to be targeted, in particular in AFR, showing a reduction of approximately 30% (table 1). The AFR estimates accounting for previous LF/OV MDA fell to 141.8 million (80.9–186.2), 102.9 million (79.6–191.2), and 79.6 million (42.7–112.2) for the three intervention thresholds (table 1 and figure 1).

Treating people infected by *L. loa* with IVM could cause severe side-effects; therefore, we estimated the population to eventually cover with MDA after excluding *L. Loa* endemic countries. The new estimation only affected African countries, because *L. loa* is only endemic in this continent. The new AFR's estimates reduced to 104.9 million (59.7–138.1), 76.1 million (58.7–141.7), and 58.7 million (31.5–82.9) for 10%, 15% and 20%, respectively. These estimates showed a further mean reduction of approximately 30% compared to the estimates made by accounting for previous LF/OV MDA (table 1 and figure 1).

## 4. Discussion

In this work, we provide the estimates of the number of individuals over 15 years of age who would need PC with IVM for the control of strongyloidiasis. The total number is huge, between 512.1 (95% CI 276–719.4) and 905.4 (95% CI 520.6–1177.2) million people, depending on the threshold of prevalence chosen to recommend the intervention, which has not yet been established. SEAR is the region with the highest numbers, followed by WPR and AFR. As expected, based on demographic reasons (i.e. the previous analysis included children from 5 to 15 years of age only [13], here we consider all people over 15 years of age), the number of adults is considerably (almost seven times) higher than the number of SAC who would require PC for strongyloidiasis, that was previously estimated between 138.9 (95% CI 74.9–195) and 245.4 (95% CI 141.2–318.7) million children, considering the 20% and the 10% threshold prevalence, respectively [13]. Compared to the previous analysis, SEAR confirms the region that would require to cover the highest number of individuals, with India, Indonesia and Bangladesh being the countries that would be most involved. By contrast, here estimates for AFR and WPR are similar, while in the previous analysis the number of SAC requiring PC was substantially higher in AFR than in WPR.

Therefore, MDA targeting both SAC and adults would entail substantially increased costs compared to PC for SAC only, owing to the higher number of people to be covered, and further expenditure would be needed to set up an infrastructure that could be used for MDA, in contrast to the already existing infrastructures available for school-based administration. Moreover, it should be considered that, in any case, the integration of strongyloidiasis into the control programmes for the other STH would require additional efforts mostly owing to the need of different diagnostic tests which may also require different biological samples/collection procedures [19]. Faecal-based methods specific for *S. stercoralis* (Baermann method and agar plate culture) [19] would probably be the diagnostic tools recommended in the WHO guidelines, but serology (with collection of dried blood spots on filter paper) might remain an option, if any commercial assay would satisfy requirements present in the upcoming target product profile and would be pre-qualified with the WHO [20].

Pending the finalization of the WHO guidelines, it is not unfounded to consider extending strongyloidiasis control programmes to adults, since older age groups have higher prevalence of this chronic infection, which is not cleared without specific treatment owing to the auto-infective cycle of the parasite. Furthermore, it should be considered that all infected people are at risk of disseminated strongyloidiasis, in case of immunosuppressant conditions/treatments [21]. Moreover, a population-based approach is expected to achieve a more rapid and deep decrease of the prevalence of infection in the general population than a school-based PC approach [12] (though there is no direct evidence for this).

The efforts for the integration of strongyloidiasis in control programmes for other neglected tropical diseases would produce additional benefits beyond the control of *S. stercoralis* infection. First, the co-administration of IVM and albendazole showed in many settings higher efficacy than benzimidazoles alone against *Trichuris trichiura*, for which the current PC strategy has demonstrated lower efficacy [22]. Moreover, scabies and other ectoparasitoses can also benefit from IVM distribution [23]. These are additional points that can influence the decision of governments on the control strategy to be implemented. Indeed, the upcoming WHO guidelines for the control of strongyloidiasis, along with these estimates, are meant to help endemic countries in the evaluation of costs and benefits of a control strategy for strongyloidiasis that also depends on available funds and other resources. On a positive note, in the last few years two generic IVM products have pre-qualified with the WHO: an asset for an easier and cheaper access to this essential drug [24].

The main limitation of this work is that the given estimates could not be calculated at sub-country level because the strongyloidiasis prevalence used was only provided at the country level. Thus, the resulting figures may overestimate the proportion of adults to be covered in a PC MDA. Another important limitation is the use of prevalences estimated for the year 2017 [8]. Although we do not believe that major changes have occurred since, changes in population demographic characteristics, human behaviour and sanitation may have affected the transmission of the parasite during the last 5 years. Moreover, owing to the large uncertainty of the prevalence of strongyloidiasis used in our study, our results showed wide CIs.

Given all these reasons, our estimates can be used for an initial evaluation of possible resources needed for PC programmes for strongyloidiasis. However, local surveys will be required to confirm these estimates. Moreover, countries should assess prevalence at district/sub-district level to decide on whether control strategies should be implemented in specific areas or country-wide.

## 5. Conclusion

In conclusion, WHO guidelines for control programmes for strongyloidiasis are under definition, and the strongyloidiasis prevalence threshold(s) for the implementation of the PC interventions will be defined. In the meantime, our estimates, complementing our previous estimates for children, can provide some basis to endemic countries that wish to start with these programmes, in order to understand costs and benefits of the extension of PC with IVM to adults.

## Data Availability

All data are included in the manuscript, table and electronic supplementary files [25].
